# Chilling the Feet and Its Consequences

**Published:** 1890-02

**Authors:** 


CHILLING THE FEET AND ITS CONSEQUENCES.—Dr. Munde says that to the imprudent act of getting out of bed without protecting the feet—one so commonly committed by women without thought of the consequences—may be traced many an attack of cellulitis, brought on by the sudden though momentary exposure of the feet to cold. It has caused more diseases to women previously healthy than could result from any other single act of imprudence.—*Medical Standard.*

